# Looking at the Ebbinghaus illusion: differences in neurocomputational requirements, not gaze-mediated attention, explain a classic perception-action dissociation

**DOI:** 10.1098/rstb.2021.0459

**Published:** 2023-01-30

**Authors:** Robert L. Whitwell, Mehul A. Garach, Melvyn A. Goodale, Irene Sperandio

**Affiliations:** ^1^ Department of Psychology, University of Western Ontario, London, Ontario, Canada N6A 5C2; ^2^ Division of Orthopaedics, St Michael's Hospital, University of Toronto, Toronto, Ontario, Canada M5B 1W8; ^3^ Department of Psychology and Cognitive Science, University of Trento, Rovereto (TN) 38068, Italy

**Keywords:** visual illusions, visual perception, grasping, eye movements, gaze, two visual systems hypothesis

## Abstract

Perceiving and grasping an object present an animal with different sets of computational problems. The solution in primates entails the specialization of separate neural networks for visual processing with different object representations. This explains why the Ebbinghaus illusion minimally affects the grasping hand's in-flight aperture, which normally scales with target size, even though the size of the target disc remains misperceived. An attractive alternative account, however, posits that grasps are refractory to the illusion because participants fixate on the target and fail to attend to the surrounding context. To test this account, we tracked both limb and gaze while participants made forced-choice judgments of relative disc size in the Ebbinghaus illusion or did so in combination with grasping or manually estimating the size of one of the discs. We replicated the classic dissociation: grasp aperture was refractory to the measured illusory effect on perceived size, while judgments and manual estimates of disc size were not. Importantly, the number of display-wide saccades per second and the percentage of total fixation time or fixations directed at the selected disc failed to explain the dissociation. Our findings support the contention that object perception and goal-directed action rely on distinct visual representations.

This article is part of a discussion meeting issue ‘New approaches to 3D vision’.

## 1. Introduction

**Figure 1.**
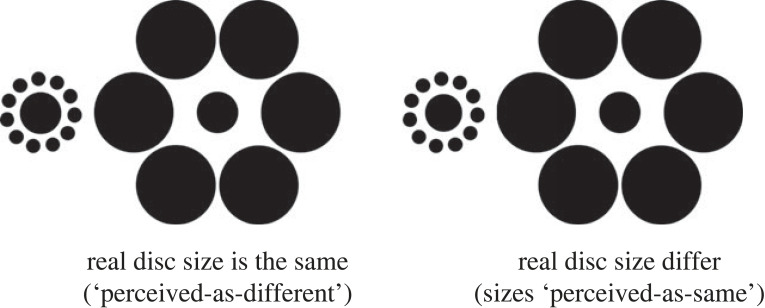
The Ebbinghaus display devised for the current study. Inner black-filled circles are surrounded by an annulus of small or large black-filled circles. Two versions of the display are illustrated, one in the left panel and one in the right, and each display is presented as a ‘dual’ configuration. Left panel: the traditional presentation in which the real sizes of the inner discs are the same but are misperceived as different. This is the critical feature of the display on ‘perceived-as-different’ trials. Right panel: a version in which the real sizes of the inner discs are different, leading to the impression that they are the same size. The difference in size required to achieve this varies from person to person, but it is scaled here to the most common difference in the study (2 mm). A pair of discs whose real sizes were different to make them appear to be the same in size is the critical component of the ‘perceived-as-same’ display.

In a now classic grasping paradigm introduced in 1995, Aglioti *et al*. [1] showed that the scaling of grasp aperture when reaching for a disc embedded in an Ebbinghaus display (figure 1) was largely refractory to the disc's misperceived size. In their experiment, participants chose one or the other disc to reach for and pick up based on a rule; half of the participants were asked to select the disc on the left side of the display if the sizes of the discs looked the same and to select the disc on the right side of the display if the sizes of the discs looked different; the rule's display-side contingency was, of course, reversed for the remaining half of the participants. Crucially, an initial two-alternative forced-choice (2AFC) test session established the difference in real disc size required to make the sizes of two discs look the same. In this session, different-sized discs were paired in the display and the participant was asked to report whether they looked the ‘same’ or ‘different’ in size [1]. Aglioti *et al*.'s [1] experiment showed, not surprisingly, that disc choice, as expressed by which of the two discs was picked up, was based on illusory disc size. Yet at the same time, the hand's in-flight grasp aperture remained tuned to the target's real size.

This dissociation was later replicated, using additional controls for visual and haptic feedback: the grasps were planned with vision available but executed without it [2]; and an additional task was introduced in which participants estimated the size of the selected disc by opening their thumb and index finger to create a matching-sized gap that was measured using the same motion-tracking techniques used for recording the grasps. Insofar as this 'manual estimate' is a faithful ‘read-out’ of the participant's visual experience of disc size, then the task permits a more direct measure of perceived size. These early studies support the view that object-vision for perception and actions rely on distinct neural representations in different networks of neural circuitry [3–7].

This interpretation has been contested by several groups for a host of different reasons (for a review, see [8]). Here, while controlling for a list of confounds (e.g. [9]), we address the earliest of these critiques [10,11], which was based on an asserted difference in the deployment of attention across the decision-making and movement execution phases of the task. We refer to this as the 'attention account', but it relies more on gaze as a proxy for selective attention.

The attention account highlights the 2AFC component, which by definition introduces a pre-grasp decision-making phase to the task. According to this account, participants saccade between each side of the dual display during this phase, fixating on one disc or the other to determine whether their sizes look the same or different. Insofar as foveated (and perifoveal or central visual) areas of the display interact with selective attention, the latter will operate on both discs and their surrounding inducers, fostering primary and secondary sources of influence on the discs’ perceived sizes: first, the effect of each inducer on the perceived size of its disc; and second, an enhancement of these single-configuration effects via a size-contrast comparison of the two perceived sizes. The resulting effect on perceived disc size is stronger than the effect of each inducer alone [10,12].

Just as important to the attention argument is the grasp execution phase after one of the disc's has been selected. During the reach, gaze is assumed to fall preferentially on the target disc. This is believed to facilitate close monitoring of the end of the reach when the hand contacts the disc and forms its grip around it (e.g. [13–16]). Thus, gaze should remain on the targeted disc-inducer configuration, to the exclusion of the other. This rationale extends to selective attention: the influence of the excluded disc and its inducer decays and so too does the size-contrast based enhancement of the illusion, leaving only the weaker single-configuration based effect to influence the hand's in-flight aperture.

For adherents to the attention-based account, the solution is a methodological one in which only one inducer and disc should be displayed per trial. This move is made in order to remove the enhanced, secondary effect of the dual display altogether, leaving the primary effect of the single configuration display intact. Using the single configuration, these authors find that the effects of the illusion on perception and grasping are equivalent [10,11,17].

An alternative method was later developed using dual-configured displays of the Muller-Lyer, Ponzo and Wundt–Jastrow illusions, in which participants reach for (or estimate the sizes of) both illusory 3D targets using their left and right hands, with some authors replicating the perception-action dissociation [18,19] and others failing to do so when visual feedback is not available throughout the reach [20]. This design reasonably assumes that participants saccade between both targets when reaching for them or estimating their sizes, and that comparisons between the targets promote a synergistic, secondary, effect involving both inducers that would register in both limbs if they were susceptible to it [20].

Thus far, there is no controversy over whether selective attention influences the different effects of the single versus dual displays on visual perception. From the perspective of *scale attention* [21], reducing the dual configuration to a single configuration removes the higher structural levels (larger scale features) in the display that are normally available for selective attention to operate on in service of scene- and ensemble-perception, which we hold as core functions of the visual perceptual system. Without the higher-level structural features of the dual display, enhancement from the secondary sources is not possible, leaving only the primary (inducer and its inner disc) sources to influence perceived size.

Nevertheless, as far as we are aware, the attention account makes no claims about gaze or the deployment of attention when participants manually estimate the size of the chosen disc. In order to explain why grasp aperture resists the dual display while the manual estimates of disc size do not, the attention account must group manual estimation and size-comparative judgements together. This account compels its advocates to make the straightforward prediction that gaze, as a proxy for selective attention, switches between the two configurations more often during manual estimation than it does during grasping—and by doing so maintains (or iteratively re-kindles) the enhanced effect of the dual display.

Our position, speculative as it is, stands in contrast to that of the attention account. According to our view, the manual estimation task requires participants to focus on one target disc to the exclusion of the other in order to reliably reproduce the perceived size of the target disc. This account predicts that gaze should behave similarly across the grasping and manual estimation tasks, particularly during the response. Specifically, gaze will be largely directed at the target to facilitate extraction of its size in order to respond accordingly.

The current experiment was designed to test these ideas, including the assumptions about gaze patterns across different tasks. Briefly, we asked participants to make judgments of relative disc size across four different tasks, two of which assumed a 2AFC format. As was done in Aglioti *et al*. [1], we first determined for each participant the difference in the discs' real sizes required for a perceived-as-same judgment using a verbal ‘same/different’ 2AFC task. We refer to this difference as the *a priori* effect of the illusion, and it is based on the display in which the sizes of the discs look the same. Next, we tracked gaze in three additional tasks while using this different-sized pair of discs to set up two versions of the display: one in which the discs looked the same size (i.e. the ‘perceived-as-same’ display); and a second in which either two of the smaller discs or two of the larger ones were used so that the sizes of the discs looked different (i.e. the ‘perceived-as-different’ display). In the remaining three tasks, participants either made 2AFC keypress judgments of the relative size of the discs, reached for the discs to pick them up, or manually estimated their sizes. In the latter two tasks, we tracked the hand using a motion-capture system. The 2AFC keypress judgments allowed us to observe baseline ‘decision-making’ preparatory patterns of gaze.

In line with the attention-based account during the decision (*preparatory*) phase of the response, we expected gaze to operate similarly across the three tasks. During the *execution* phase, however, we expected gaze would cluster more, in terms of number and time, towards the chosen side of the display for both the grasps and manual estimates. Meanwhile, the effect of Ebbinghaus display was predicted to dissociate across the two tasks. Thus, our account emphasizes the behavioural endpoints of the task and the operations that support them. In short, it is what must be done with selected target information that matters most [4,22].

## 2. Material and methods

### (a) Participants

Twenty-four adult participants (mean years of age was 21; 14 female) recruited from the student population at The University of Western Ontario took part in the study. All were naive to the purpose of the experiment. Participants self-reported right-handed with normal- or corrected to normal vision, and written informed consent was obtained prior to testing. Furthermore, they received monetary compensation for their time. All procedures were approved by the Research Ethics Board of The University of Western Ontario and were carried out in accordance with the *Tri-Council Policy Statement Ethical Conduct for Research Involving Humans* [23].

### (b) Stimuli and apparatus

The workspace was comprised of a desk with a chinrest and an LCD monitor (which was used to calibrate the eye-tracker) tilted approximately 10° from the horizontal. The monitor was tilted in order to provide a more comfortable view for the participant while approximating a perpendicular viewing angle for the head-mounted eye-tracker. For the experimental tasks, the Ebbinghaus display was placed on top of the LCD screen.

As shown in figure 1, the display consisted of a white board (38 cm wide × 22.5 cm in height) with two annuli of matte black-filled circles (*inducers*): one to the left of the display and one 13 cm to its right. Each inducer was centred at the midpoint of the display's height (approx. 11.3 cm). For the display as arranged in figure 1, the centre of the inner disc on the left was 10.5 cm from the display's leftmost edge, whereas the centre of the inner disc on the right was 14.5 cm from the display's rightmost edge. The inducers on one side of the display comprised an annulus of 11 black-filled circles each 1 cm in diameter. Each of these small circles were spaced evenly apart from their immediately neighbour and centred 2.5 cm from the centre of the ring. The inducer on the other side of the display comprised an annulus of six black-filled circles each 5.8 cm in diameter. These large circles were spaced evenly apart from their immediate neighbours and centred 6 cm from the centre of the inducer. Real 3D discs were positioned at the centres of each inducer, creating an illusion of size. The discs were from two sets of five discs each 3 mm thick and painted matte black. The smallest disc used in each set was 3 cm in diameter while the largest was 3.5 cm.

An OptoTRAK optoelectronic motion-tracking system (NDI, Waterloo, Canada) was used to track hand movement at 100 Hz. An Eye-Link II (SR Research Ltd., Mississauga, Canada) system was used to record the position of the right eye at a sampling rate of 250 Hz. A Logitech keypad was used in the keypress task to track participants' choice and response or *reaction time*, and a button-release device was used in the manual estimation and grasping tasks to track reaction time in these tasks. These were positioned on the table to the right of the display and were elevated to approximate the height of the display. One red LED light was used as a resting fixation point between trials, while a second LED light was used to illuminate the workspace to signal the start of the trial (see the electronic supplementary material, figure S1).

### (c) Experimental design

Four tasks were administered. The first of these tasks was always a verbal forced-choice (same or different) judgment of relative disc size without movement recording equipment. This was a semi-formal session used to determine the *a priori* effect of the illusion. In this session, participants were asked to say whether the disc sizes looked the same or different after each paired presentation. One of the discs was always 3 cm in size, and the size of the other disc was varied in order to find a difference that elicited a reliable ‘same’ response. The 3 cm disc and its different-sized companion, the partner disc size were specific for each participant and were presented in two ways in the remaining three tasks: first, for the perceived-as-same display, the physically larger disc was placed at the centre of the large-circles inducer, while the physically smaller disc was placed at the centre of the small-circles inducer; second, for the perceived-as-different display, the discs were either the smaller or the larger partners of the pair.

Following the initial verbal 2AFC session, three tasks, 2AFC *keypress*, *grasping* and *manual estimation*, were administered in a random order to each participant in a blocked-trial format. Eye-tracking was performed for these three tasks, while hand movement tracking was performed in the grasping and manual estimation tasks only. Nine of the participants, selected at random, were provided with the following response rule: ‘if the sizes of the centre discs look the same, choose the disc on your left; if the sizes of the centre discs look different, choose the disc on your right’. For the remaining participants, the rule merely reversed the left/right contingency. In order to minimize rule confusion, each participant applied only one version of the two rules throughout the experiment. The display was flipped 180° on half of the trials in a randomly interleaved format in order to minimize the possibility that participants would explicitly work out the real sizes of the discs or, through motor learning or adaptation, calibrate their grasp aperture to their real sizes [24–27], (see the electronic supplementary material for additional details).

In the grasping and manual estimation tasks, the participants expressed their judgement verbally: ‘same’ for discs judged to look the same in size; and ‘different’ for discs judged to look different in size. This was done to preserve the verbal component for these two tasks and to make the disc choice for the manual estimation task explicit. In the grasping task, participants reached for the chosen disc to pick it up and put it back down. In the manual estimation task, participants indicated the perceived size of the chosen disc by opening their index finger and thumb to create a matching-sized gap, the *manual estimate aperture*. In both the grasping and manual estimation tasks, participants were encouraged to make their verbal judgment before initiating their hand movement. In general, participants adhered to this instruction. However, without voice recording to make a formal evaluation, it is possible their brief ‘same’/‘different’ verbal response overlapped with movement initiation on some trials.

Each task comprised 24 trials. This number reflected a trade-off between sample stability and the participant's well-being, as we discovered during piloting the project that the head-mounted eye-tracking unit was cumbersome for some participants, whose discomfort increased over time. The 24 trials for the each of the keypress, grasping and size-estimation tasks were organized into 12 perceived-as-same trials and 12 perceived-as-different trials. On six of the 12 perceived-as-different trials, the smaller partner of the disc pair was presented in both inducers, and on the remaining six trials, the larger partner was presented in both inducers. With board-orientation flipping, the target was on the left for six of the 12 perceived-as-same trials and six of the 12 perceived-as-different trials, and on the right for the remaining six trials of these two trial types. The presentation order of these conditions was randomized.

### (d) Procedure

The verbal judgment task was administered using a staircase method to determine the *a priori* effect (see the electronic supplementary material for additional details). Participants were presented with pairs of different-sized discs and asked to say whether they looked the same or different. The experimenter began with no size difference and increased the size difference incrementally on each trial by keeping one disc the same size and altering the size of the other. After a ‘same’ response was elicited, the experimenter started with the maximum difference in disc size (5 mm) and reduced it incrementally on each trial in a similar manner as described above.

After the initial session, the Eye-Link II was calibrated using its native nine-point procedure before the start of each of the three remaining tasks. For the three tasks, each trial began with an auditory cue for participants to fixate on a red LED light located above the illusory display. Not long after, a second auditory cue coincided with the illumination of the workspace and cued participants to scan the display, make and announce their choice (in the case of the manual estimates and grasps) and then execute their response (see the electronic supplementary material, figure S1).

For the grasping and manual estimation tasks, participants held down a button on a portable button box with their right index finger and thumb pinched together. Participants were instructed that the button was the hand's start and end location for these two tasks. The keypad for the keypress task was positioned at approximately the same location as the button box.

In the grasping task, participants reached for their chosen disc, lifted it up using their index finger and thumb, and put it back down before returning to the home position. In the manual estimation task, participants spread their thumb and index finger apart to create a gap that matched the disc's perceived size. The participants were also instructed to refrain from reaching for the disc in this task. After they were finished with their estimate, they returned to the start button at which point the illuminator was switched on again, cueing the participants to reach for the previously chosen disc. This allowed the participants the same haptic feedback about the disc's real size that was available for the grasping task. After this, the participants returned their hand to the start button in preparation for the next trial. In the keypress task, participants indicated their disc choice by keypress on the Logitech keypad.

### (e) Eye and hand tracking

Eye movements were recorded for 5 s from the onset of each trial.

The 3D positions of the right hand were recorded using an OPTOTRAK system (Northern Digital, Waterloo, Ontario, Canada) sampling the two infrared emitting diodes (IREDs) on the participants hand at a rate of 100 Hz for a minimum of 5 s from the onset of each trial.

### (f) Data pre-processing

The limb kinematic data comprised 3D positions of each IRED for each sample frame. These data were pre-processed and analysed in a standardized way to extract a number of kinematic landmarks (e.g. [9], see the electronic supplementary material for additional details). Grasp aperture was computed as the distance between the index finger and thumb IREDs at each sample frame. The principal dependent measure for the grasps was peak grasp aperture (PGA), which was defined as the largest aperture achieved from the start of the movement to the end of the reach.

Like grasp aperture, the *estimate aperture* was computed as the difference between the index finger and thumb IREDs at each sample frame for the manual estimation task. The final estimate aperture (FEA) was determined as the point at which the estimate aperture's rate of change stabilized around zero, signalling that a relatively consistent estimate had been achieved (see the electronic supplementary material for the definition and analysis of the peak estimate aperture, which did not meaningfully influence the estimates of the effect of the illusion).

Trials in which participants gave an ‘incorrect’ judgment (e.g. a ‘same’ judgment when discs that should have been perceived as different were displayed) were removed from the analysis (8% of trials). This left a uniform sample of trials (92%) in which disc choice accorded with the intended effect of the illusion. Furthermore, all fixations shorter than 80 ms in duration were discarded, as this corresponds to the minimum duration for higher-level processing of visual information to occur (e.g. [28]). Fixations that fell outside the display area were also removed.

We applied cluster analysis to the gaze data (*x-* and *y*-values in units of screen-width and screen-height pixels, respectively) to identify, in a data-driven manner, four clusters corresponding to the four positions of the target inner disc (across trials), arranged left to right and to assign each fixation to one of these four clusters. With the display oriented as depicted in figure 1 (i.e. the small inducer on the left side), the clusters corresponding to the two configurations were the leftmost and third cluster from the left. With the display flipped 180° (i.e. the large inducer on the left side), the corresponding clusters were second from the left and the rightmost cluster. Assigning fixations to each cluster allowed us to quantify the following dependent variables: (i) the number of display-wise saccades per second, which was defined as the number of instances in which temporally adjacent fixations left one cluster for another where the second cluster skipped one or two adjacent clusters. For example, suppose gaze falls at the leftmost cluster and then falls on either the third cluster from the left or the rightmost one, then this transition would be counted as a display-wide saccade; (ii) the number of fixations on the target disc. Note that the target disc was the disc chosen in accordance with the illusion; (iii) the fixation time on the target disc; (iv) the percentage of fixations on the target disc (out of the total number of fixations); and (v) the percentage of fixation time spent on the target disc (as a percentage of the total number of fixations).

For the fixation (gaze) data, the button-release time (the reaction time, RT) was used to separate the preparatory and execution phases. For the grasps, estimates, and keypress tasks, the preparatory phase began when the illuminating light was turned on. The preparatory phase ended when the button was released for the grasps and estimates, or when the keypress was made during the keypress task. The execution phase for the grasps and manual estimates began with the release of the start button. For the estimates, the end of the execution phase was defined as the point in time at which the FEA was achieved. For the grasps, the end of the execution phase was defined as the point at which the reach ended. The keypress task was considered to be comprised of preparatory time only.

### (g) Statistical analysis

For each combination of participant, task and dependent measure, means were computed from trials grouped into combinations of the target disc's size (small versus large) and surrounding inducer (small or large) and whether the display was set up to make the sizes of the inner discs look the same or different (i.e. *display type*).

For the perceived-as-different display, the effect of the illusion was computed for the grasps and manual estimates as the mean dependent measure when the target disc was surrounded by the small inducer (i.e. when the disc was perceived as larger than its actual size) minus the mean dependent measure when the target disc was surrounded by the large inducer (i.e. when the disc was perceived as smaller than its actual size). Positive difference values therefore reflect the expected effect of the illusion. Note that for the perceived-as-different trials, target disc size varied independently of the surrounding inducer, and so the effect on each disc size in this condition was collapsed for brevity and to boost sample stability. For completeness, *t*-tests for an influence of disc size on the effect of the display in this condition were null for the grasps, *t*_18_ = .34, *p* > 0.73 and for the manual estimates, *t*_18_ = 0.95, *p* > 0.35.

The effect of the perceived-as-same display requires one additional step, because the above-mentioned difference formula would yield a value of zero for the estimates and the grasps under the null hypothesis; recall that the sizes of the discs are perceived to be the same and that, according to the null hypothesis, both the estimates of disc size and the grasp aperture for the grasps are faithful ‘readouts’ of perceived size. The solution, for us, was to add the *a priori* effect (i.e. the difference in disc size required to make the discs look the same size) to the result of the above-mentioned formula for both the grasps and the manual estimates (see the electronic supplementary material for additional details). Thus, the interpretation of the results for both display arrangements are comparable: positive values nominally represent the effect of the illusion.

To test the mean effects of the illusion, a repeated-measures ANOVA was performed with the following factors: *task* (keypress, grasps, and manual estimates) and what we refer to as *display type* (the ‘perceived-as-same’ and ‘perceived-as-different’ arrangements). Type I error was set to 0.05 for each ANOVA. For each family of follow-up contrasts, we used the Holm step-down procedure [29] in combination with the moderately more powerful multiplicative Bonferroni inequality [30] to control the type I error (the Holm–Sidak procedure, see also [31,32]). This procedure sequentially adjusts the per-contrast alpha for a given family of tests while holding the type I error at 0.05 on a per-family basis; it is more powerful than the more commonly used Bonferroni procedure, yet maintains much of the latter's favoured simplicity.

### (h) Unadjusted and adjusted effects of the illusion

Response sensitivity to differences in target size should be matched across tasks. When they are not, the difference can lead to spurious differences in effects across tasks when none exist. This holds for the effects of illusions across tasks [33]. A linear function is a good approximation of the relationship between PGA (or the FEA) and target size. The crucial parameter is the coefficient relating differences in mean response to incremental changes in target size (i.e. the *slope*). The mean slopes indicate that the unadjusted effect of the illusion is overestimated for the manual estimates and underestimated for the grasps (slope for the manual estimates: *M* = 1.15, s.e.m. = 0.104, *t*_18_ = 11.12, *p* < 2 × 10^−9^, slope for the grasps: *M* = 0.84, s.e.m. = 0.102, *t*_18_ = 8.45, *p* < 2 × 10^−7^). Moreover, the mean slopes differ significantly, *t*_18_ = 2.44, *p* < 0.03. Crucially, the mean illusory effect can be adjusted to compensate for this difference. This is done by dividing the mean unadjusted effect by the mean slope. However, this procedure neglects the variance of the slope, leaving the variance of the ratio unspecified [34]. Fieller's theorem [35] provides exact confidence intervals for the ratio of two random variables, and a Taylor approximation has been shown to perform well when the slopes differ highly significantly from zero [36], as is the case here. The Taylor approximation is advantageous as it can be rearranged to provide an approximation for each participant, which is useful for performing statistical procedures on samples [37]. Thus, we adjusted the effects of the illusion on the grasps and manual estimates using this method, as recommended by Franz and colleagues (e.g. [36,37]). This adjustment did not affect the conclusions.

## 3. Results

### (a) *A priori* effect size and choice accuracy

The mean difference in real size that resulted in reliable ‘same’ size judgements was 2.6 mm (1 –4 mm). Overall, on average, 92% of participants' choices matched the expected effect of the display. Furthermore, this level of performance was not modulated by task (*F*_2,34_ = 0.28, *p* > 0.74), the display type (*F*_1,18_ = 2.48, *p* > 0.13) or the interaction effect, *F*_2,33_ = 2.3, *p* > 0.12.

### (b) Reaction times (preparatory phase)

**Figure 2.**
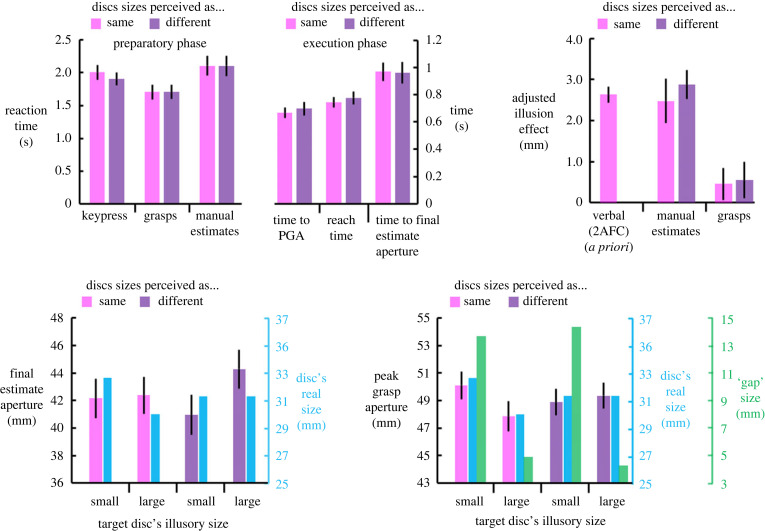
Top left: Preparatory and execution phase temporal dependent measures as a function of task and display type. The grasps were initiated the fastest, and the mean initiation time for the estimates and the mean keypress time for the keypress task were statistically equivalent. With respect to the execution phase, the reach time for the grasps was faster than the FEA time. Top-right: the *a priori* effect of the Ebbinghaus illusion, using the ‘perceived-as-same’ display (established in the initial verbal 2AFC task), and the adjusted effects of the Ebbinghaus illusion on the manual estimates (middle) and grasps (right) as a function of display type. The effect of the illusion on the estimates was comparable to the *a priori* effect, whereas the effect of the display on the grasps was null. Bottom panels: FEA for the manual estimates (left) and PGA for the grasps (right) as functions of the direction of the effect of the illusion (small–the inducer makes the target disc appear smaller than its real size; large–the inducer makes the target disc appear larger than its real size) and the display type (i.e. the perceived-as-same and perceived-as-different arrangements). The blue bars depict real-disc size for the ‘perceived-as-same’ display, whereas they depict average disc size for the ‘perceived-as-different’ display. The average is used for real-disc size (and for the illusion effect) because the two are independent of one another for these trials. Note that the blue bars possess their own ordinate axis to the right of each panel. The green bars in the bottom right panel reflect the mean distance between the outside edge of the target disc and the inner-most edges of the surrounding inducer. The green bars possess their own ordinate axis to the right of this panel. Importantly, while all ordinate axes possess different minimum and maximum values, they share the same units and span the same distance (12 mm). The figure indicates that the illusion drives the estimates aperture but that real-disc size drives the PGA. Error bars are s.e.m. (Online version in colour.)

The top left panel of figure 2 (preparatory phase) shows mean RT as a function of task and display type. Mean RT was influenced by task (*F*_2,32_ = 5.72, *p* < 0.01, $\eta_{p}^{2}=0.24$), but not by the display type, *F*_1,18_ = 0.35, *p* > 0.55. The interaction effect was null, *F*_2,27_ = 2.47, *p* > 0.11. The grasps were faster to initiate than either the manual estimates (*t*_18_ = 3.44, *p* < 0.003) or the 2AFC keypress responses (*t*_18_ = 2.58, *p* < 0.02); the RT for the latter two did not differ statistically, *t*_18_ = .93, *p* > 0.36.

### (c) Grasp and estimate temporal measures (execution phase)

The top left panel of figure 2 (execution phase) shows the time to peak grip aperture (PGA) and reach completion time (reach time) for the grasps, and the time to FEA for the manual estimates. These measures varied with task (*F*_1,18_ = 63.56, *p* < 3 × 10^−7^, $\eta_{p}^{2}=0.78$) but not with display type (*F*_1,18_ = 0.43, *p* > 0.51). The interaction was null, *F*_2,19_ = 0.87, *p* > 0.36. Pairwise comparisons were all significant (all *p*s < 0.007), such that the time to PGA occurred earliest, followed by the time to complete the reach, and then time to complete the estimate of target size.

### (d) Illusory effects on grasp aperture and final estimate aperture

The effect of the Ebbinghaus illusion established during the initial verbal 2AFC task, an effect we termed the *a priori* effect, is shown in the top-right panel of figure 2. To the right of the *a priori* effect are the adjusted effects of the illusion on the FEA of the manual estimates (henceforth *manual estimates*) and on the PGA (henceforth *grasps*), respectively (see the electronic supplementary material, figure S3 for both the unadjusted and adjusted illusory effects). This panel bears three findings that are supported more formally below: (i) the similarity of size of the illusion effect in the two perceptual tasks: the verbal 2AFC and the manual estimates in the perceived-as-same display; (ii) the difference between these perceptual effects and the null effect on the grasps for this display; and (iii) the replication of these two points for the perceived-as-different display.

The effect of the illusion on the manual estimates did not vary as a function of display type, *t*_18_ = 0.93, *p* > 0.36. An analogous test applied to the grasps was also null, *t*_18_ = 0.16, *p* > 0.87. For the perceived-as-same display, the *a priori* effect of the illusion was significantly greater than the effect of the illusion on the grasps (*t*_18_ = 4.75, *p* < 2 × 10^−4^), but not significantly different from the effect of the illusion on the manual estimates, *t*_18_ = 0.37, *p* > 0.71. Furthermore, the effect on the manual estimates was significantly greater than the effect on the grasps, *t*_18_ = 3.11, *p* < 0.006. For the perceived-as-different display, the effect of the illusion on the manual estimates was significantly greater than the effect on the grasps, *t*_18_ = 5.42, *p* < 4 × 10^−5^. Thus, both illusory arrangements consistently affected estimates of disc size but not the hand's grasp aperture.

The bottom panels of figure 2 reinforce the key takeaways from figure 2's top-right panel. The bottom panels depict the mean FEA (bottom left) and the mean PGA (bottom right) as functions of the direction of the effect of the illusion and the display type. Notably, regardless of the display type, the FEA tracks the perceived sizes of the discs; by contrast, the PGA tracks the real sizes of the discs.

The bottom right panel of figure 2 depicts mean PGA as a function of the display type and the illusory size of the disc. In this panel, green bars depict the sizes of the gaps between the outside edge of the inner discs and the inside edge of the inducer. This panel suggests that PGA is not influenced by the size of the gaps between the inner edges of the inducer and the outer edge of the inducer's inner disc. According to one account of the perception-action dissociation, the inducers are treated by the visuomotor system as obstacles the fingers must avoid when reaching for the target disc [38,39]. This avoidance translates into an influence on PGA ([39,40], cf. [26,41]). The original formulation of this idea suggested that the narrower the gap, the smaller the PGA. Furthermore, because gap size is often smaller for the small inducers than for the large ones and because the small inducers increase the perceived size of the disc (whereas the large inducers decrease the perceived size of the disc but typically possess a larger gap), the obstacle-avoidance system is thought to operate in opposition to the effect of the illusion, resulting in a reduced or null effect on grasps only. According to this account, the obstacle-avoidance system masks a real effect of the illusion on the grasps.

A formal test for influence of gap size on grasp aperture was couched in terms of whether the reduction in gap size that occurs moving from the large inducers to the small ones affected PGA *consistently* across the ‘perceived-as-same’ and ‘perceived-as-different’ displays. In line with the obstacle-avoidance account, PGA was significantly smaller for the small gap than for the large one for the perceived-as-same display, *t*_18_ = 6.22, *p* < 8 × 10^−6^. Crucially, however, PGA did *not* vary as a function of the reduction in gap size for the perceived-as-different display, *t*_18_ = −1.35, *p* > 0.19. Thus, the reduction in gap size failed to reduce PGA across both display types. Since the illusion did not influence PGA across both display types, we are left with the real difference in size as the lone explanatory factor for both displays.

Overall, the analysis of PGA shows that this measure tracks the real sizes of the discs, rather than the perceived size or the size of the gap between the outside of the disc and the inner edges of the surrounding annuli. The manual estimates, in stark contrast, track the perceived sizes of the discs.

### (e) Fixations

Table 1 lists the five fixation-based measures, the ANOVA tests of the main effect of response type, and follow-up pairwise comparisons for the preparatory time frame and then the preparatory and execution time frames combined.

**Table 1.** Fixation measures, ANOVA tests performed on them (d.f._1_/d.f._2_ = 2/36, then Greenhouse-Geisser adjusted), and subsequent pairwise comparisons (d.f. = 18). (Bold entries indicate statistically significant tests.)

measure and ANOVA statistic	response phase	pairwise *t*-test statistics	
number of display-wise	preparatory	G > KP:	*t* = 0.34, *p* > 0.73
saccades per second		**KP > M:**	***t* = 5.25, *p* < 6 × 10^−5^**
*F* = 16.83, *p* < 3 × 10^−5^, $\eta_{p}^{2}=0.48$		**G > M:**	***t* = 5.42, *p* < 4 × 10^−5^**
execution		G > M:	*t* = 1.42, *p* > 0.17
number of fixations on the	preparatory	**G > KP:**	***t* = 2.14, *p* < 0.05**
target disc		**M > KP:**	***t* = 3.94, *p* < 0.001**
*F* = 10.49, *p* < 0.002, $\eta_{p}^{2}=0.37$		**M > G:**	***t* = 2.85, *p* < 0.02**
execution		G > M:	*t* = 0.4, *p* > 0.69
fixation time on the	preparatory	KP > G:	*t* = 0.52, *p* > 0.6
target disc		**M > KP:**	***t* = 4.75, *p* < 2 × 10^−4^**
*F* = 17.27, *p* < 2 × 10^−5^, $\eta_{p}^{2}=0.49$		**M > G:**	***t* = 4.68, *p* < 2 × 10^−4^**
	execution	**M > G:**	***t* = 2.34, *p* < 0.04**
fixations on the target	preparatory	**G > KP:**	***t* = 3.79, *p* < 0.002**
disc as % of total		**M > KP:**	***t* = 6.4, *p* < 6 × 10^−6^**
*F* = 21.99, *p* < 1 × 10^−6^, $\eta_{p}^{2}=0.55$		**M > G:**	***t* = 3.06, *p* < 0.007**
execution		M > G:	*t* = 0.12, *p* > 0.9
fixation time on the target	preparatory	G > KP:	*t* = 1.61, *p* > 0.12
disc as % of total		**M > KP:**	***t* = 6.77, *p* < 3 × 10^−6^**
*F* = 24.89, *p* < 1 × 10^−6^, $\eta_{p}^{2}=0.58$		**M > G:**	***t* = 4.58, *p* < 3 × 10^−4^**
	execution	G > M:	*t* = 1.49, *p* > 0.15

**Figure 3.**
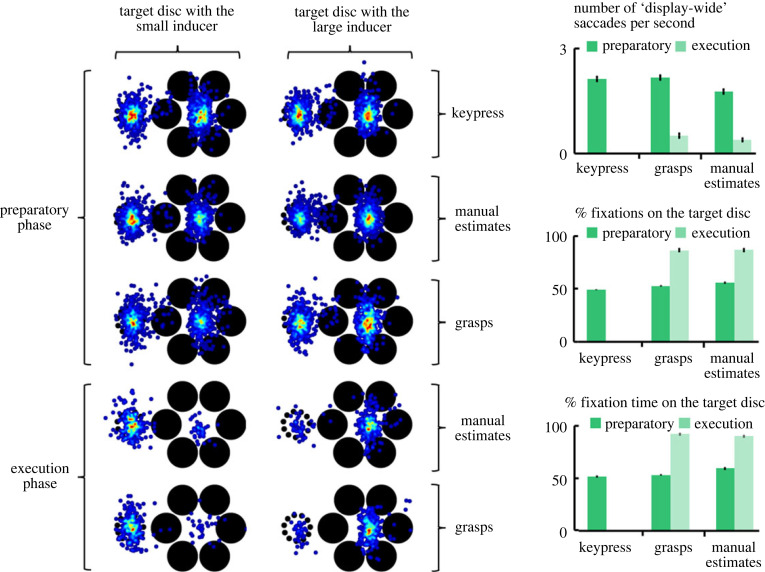
The spatial distribution of fixations and the fixation measures as functions of task and the response phase (i.e. the preparatory and execution phases). Left: gaze maps show the spatial distribution of fixations parsed into trials in which the target disc was surrounded by the small or large inducer. Display orientation is collapsed such that fixations on those trials with the opposite board orientation to the one depicted were flipped across the central vertical axis of the full set of fixations. Note that the target disc was removed for the illustration. Top-right: the number of ‘display-wide’ saccades (gaze shifts between disc positions two or more locations apart from one another) per second. Middle right: the percentage of total fixations that were directed at the target disc. Bottom right: the percentage of total fixation time that was directed at the target disc and inducer (right). During the preparatory phase, participants made more saccades between the discs, fixated on the target disc less, and spent less fixation time on the target, relative to the execution phase. Moreover, none of the fixation measures during the preparatory phase differentiate the keypress and manual estimation tasks from the grasping task. During the execution phase, the fixation measures fail to differentiate the grasps and manual estimates. Error bars are s.e.m. (Online version in colour.)

The spatial distribution of fixations as a function of the task and the phase of the response, along with the mean fixation measures as a function of the task and the preparatory and execution phases of the response, are depicted in figure 3. Furthermore, because the reaction times varied as a function of the response type, and because the execution time differed between the grasps and manual estimates, figure 3 was limited to the time-standardized fixation measures: the number of display-wise saccades per second; the percentage of fixations in the target area (of the total number of fixations); and the percentage of total fixation time spent in the target area (see the electronic supplementary material, figure S4 for the number of fixations on the target and the fixation time on the target).

Overall, the fixation measures differentiated the tasks well. However, for the time-controlled fixation measures, the grasps and estimation tasks looked more similar to each other than they did to the keypress task. Notably, the attention account suggests that longer fixation time on the target weakens the illusion, because it reflects selective attention operating on the fixated target and its inducer to the exclusion of the other disc and its inducer. By that same token, fixations distributed across both sides of the display should strengthen the illusion, as this pattern of gaze suggests selective attention is operating on both discs and their inducers. Given that the verbal 2AFC and manual estimation tasks yielded the largest illusion effects while the grasps remained unaffected, the attention account suggests that the fixations made during the two perceptual tasks should be distributed across both sides of the display while the fixations made when grasping should be anchored to the target. Crucially, we did not find this.

Take, for example, the mean number of display-wise saccades per second. During the preparatory phase, this measure was lowest for the estimations. The attention- based account predicts that the keypress and estimates should exhibit higher mean number of display-wise saccades per second, while the grasps should exhibit the lowest mean number of display-wise saccades per second. Furthermore, the keypress and manual estimation means for the remaining preparatory phase time-standardized fixation measures were most dissimilar, with the mean values for the grasps typically falling in-between those of the keypress and estimation tasks (figure 3). Specifically, the mean fixation time on the target disc as a function of total fixation time was greatest for the manual estimates, intermediate for the grasps, and least for the keypress task (table 1); and the mean number of fixations on the target disc as a percentage of the total number of fixations was greatest for the manual estimates and least for the grasps and keypress tasks, with the latter two statistically indistinguishable (table 1). Thus, during the preparatory phase, gaze was more likely to shift between the discs and to engage the non-target disc during the keypress and grasping task than during the manual estimation task.

Turning to the execution phase, the attention-based account assumes that gaze is directed preferentially to the target during the grasp. We take no issue with this prediction. Moreover, the data support it. In fact, across all measures, gaze preference to the target increased significantly in the execution phase, relative to the preparatory phase, for the grasps and for the manual estimates (all *p*s < 2 × 10^−7^). In each of the preparatory and execution phases, gaze time on the target was significantly greater for the manual estimates than for the grasps (table 1). This result stands in opposition to the attention account, given that the manual estimates, not PGA, was affected by the illusion. Nevertheless, we find it more likely that this difference in time spent fixating the target merely stems from the increased preparatory and execution times for the estimates. This issue reinforces the importance of the measures of gaze that control for differences in preparatory and execution times. Indeed, the number of display-wide saccades per second, the number of fixations on the target as a percentage of total fixations, and the fixation time on the target as a percentage of total fixation time were each statistically indistinguishable between the grasps and manual estimates during the execution phase (table 1). Thus, the fixation analysis shows that gaze does not differentiate the two tasks in a way that can explain why the estimates faithfully expressed the misperceived sizes of the discs while the grasps expressed the real sizes of the discs.

## 4. Discussion

Contemporary theories of human vision hold as a given that the organization of the primate visual system reflects the evolution of distinct networks of neural circuitry, each broadly suited for solving unique problems posed by different facets of adaptive behaviour (for reviews, see [3,5,6,42]). Visual perception entails the moment-to-moment construction of a mental model of what is ‘out there’ to serve as a kind of cognitive sandbox in which plans and goals can be formulated and behaviourally relevant options derived, selected and acted on. One function of visual perception is visual recognition which entails classifying and semantically elaborating real-time visual sensory information with the assistance of stored representations. A core challenge for visual perception is in achieving stable object constancies: that is, to filter out the viewpoint-dependency in the array to parse it and extract the core semantic relevancies to the organism. The cognitive adjudication of numerous possibilities does not require the extraction of metric visual information. Put another way, selection for priority amongst competing possibilities could rely on relative comparisons, in which viewpoint dependencies and metric processing are discounted.

When reaching for a goal object, however, viewpoint-dependent, metric information is critical. Here, the problem entails extracting the 3D object structure and representing its egocentric relationship to the viewer for executing the limb movement required for acquiring the goal object. The grasp points on the goal object must be suited for lifting and manipulating it, the hand's in-flight grasp aperture must be large enough to accommodate the object while the arm movement must deliver the hand to it. As a consequence of these more metric requirements, neural circuitry has evolved for the visual control of object-directed actions that is distinct from that mediating the visual perception of objects [4,42]. Recent work has shown, for example, that grasp aperture and grasp angle are refractory to the mean size and orientation of an ensemble of distractors around a target object [43], and that under conditions of attentional crowding, grasp aperture remains tuned to target size while manual estimates of target size do not [44].

Evidence from a number of different lines of work supports this duplex account of visual processing. One of the most controversial pieces of evidence comes from the apparent resistance of visually guided actions to familiar pictorial illusions, such as the Ebbinghaus and Ponzo illusion (for review, see [45]). It has also been argued, however, that the dissociation is not so much a difference in the neural circuitry mediating visual perception and the neural circuitry mediating visual control of action as it is a difference in the deployment of gaze (and selective attention) when judging an object's size versus reaching out to pick it up. In the case of the Ebbinghaus illusion, it has been postulated that fixations (selective attention) deployed across the full display enhance the contrast in apparent size between the two central discs that are already influenced by their surrounding annuli. When reaching out to pick up one of the central discs, however, fixations are restricted to that disc and its immediate vicinity, thereby reducing the illusory effect. In the current study, we systematically investigated the validity of this critique.

Using the original dual display paradigm, we replicated the finding that grasp aperture is refractory to the illusion even when one disc is chosen on the basis of differences in apparent size as well as the finding that manual estimates of disc size remain biased. Importantly, we tracked gaze while participants performed these tasks. Using gaze as a proxy for attention while participants performed three tasks, namely grasps, manual estimation and a forced-choice keypress, we found that the patterns of fixations in terms of the number of display-wide saccades and the percentages of fixations and fixation time directed at the target disc and inducer, while differentiating the tasks, did not do so in accordance with the effect of the illusion. Moreover, when the response execution phase was included, the fixations when grasping and when manually estimating disc size were statistically indistinguishable. In so far as gaze serves as a spatiotemporal marker of selective attention across the visual array, our findings suggest that selective attention to the target disc cannot explain the task-dependent effect of the display.

One limitation of the current study is that we established an *a priori* effect using a ‘same’/’different’ verbal 2AFC format and we did not measure the effect of the illusion on disc size during the ‘same’/’different’ keypress version of the 2AFC task. Furthermore, gaze was not tracked for the verbal 2AFC task. However, it is important to recall that the percentage of choices guided by the illusion was statistically equivalent across the keypress, grasps, and manual estimation tasks, and that the measured effect of the illusion on the estimates was no different than the *a priori* one, which was determined from the verbal 2AFC task. Thus, it seems reasonable to assume that the illusion was operating just as strongly during the keypress task as it was during the verbal forced-choice and estimation tasks.

We conclude that the key difference lies in the distinct problems that manually estimating the size of target and grasping the target pose for the visual system—and, as a consequence, the different computations that are required.

## Data Availability

Data for the reported experiment is currently available via the Open Science Framework (OSF): https://osf.io/3ucvd/?view_only=5030d0ac14df4e55b11d228af467fe9b. The data are provided in the electronic supplementary material [46].
